# Curcumin Inhibits NTHi-Induced MUC5AC Mucin Overproduction in Otitis Media via Upregulation of MAPK Phosphatase MKP-1

**DOI:** 10.1155/2017/4525309

**Published:** 2017-04-12

**Authors:** Anuhya Sharma Konduru, Shingo Matsuyama, Byung-Cheol Lee, Kensei Komatsu, Jian-Dong Li

**Affiliations:** Center for Inflammation, Immunity & Infection, Institute for Biomedical Sciences, Georgia State University, Atlanta, GA 30303, USA

## Abstract

Otitis media (OM), characterized by the presence of mucus overproduction and excess inflammation in the middle ear, is the most common childhood infection. Nontypeable* Haemophilus influenzae* (NTHi) pathogen is responsible for approximately one-third of episodes of bacteria-caused OM. Current treatments for bacterial OM rely on the systemic use of antibiotics, which often leads to the emergence of multidrug resistant bacterial strains. Therefore there is an urgent need for developing alternative therapies strategies for controlling mucus overproduction in OM. MUC5AC mucin has been shown to play a critical role in the pathogenesis of OM. Here we show that curcumin derived from* Curcuma longa* plant is a potent inhibitor of NTHi-induced MUC5AC mucin expression in middle ear epithelial cells. Curcumin inhibited MUC5AC expression by suppressing activation of p38 MAPK by upregulating MAPK phosphatase MKP-1. Thus, our study identified curcumin as a potential therapeutic for inhibiting mucin overproduction in OM by upregulating MKP-1, a known negative regulator of inflammation.

## 1. Introduction

Mucin glycoproteins are a major component of mucus secretions in the middle ear, trachea, and digestive and reproductive tracts. Mucus production represents a protective innate defense mechanism to protect and lubricate the epithelium and trap invading pathogens for removal by the mucociliary clearance system [1]. However, in chronic infections, excess mucin impairs the mucociliary clearance system, resulting in mucus accumulation and poor function of the mucus-lined epithelial tracts. Of the ~24 mucin genes identified till date MUC5AC mucin has been shown to play a critical role in the pathogenesis of upper respiratory tract infections including otitis media (OM). OM is characteristic of the presence of mucus overproduction and excess inflammation in the middle ear [2, 3]. In patients with OM, increased mucus effusion into the ear's tympanic cavity impairs the movement of the eardrum and middle ear bones and leads to hearing problems. A higher concentration of mucin in the middle ear effusion has been shown to correlate with the extent of hearing impairment [4, 5]. While mucin upregulation is an important innate defense response of the host to infections in the middle ear, excess mucin can lead to impaired mucociliary clearance and conductive hearing loss [2]. Therefore, mucin expression must be tightly controlled.

Nontypeable* Haemophilus influenzae* (NTHi) represents the cause of approximately one-third of episodes of OM. Current treatments for bacterial OM rely on the systemic use of antibiotics, which often leads to the emergence of multidrug resistant bacterial strains [6, 7]. Development of NTHi vaccine remains a challenge due to the high genetic diversity of NTHi strains and high antigenic variability of surface-exposed antigens [8, 9]. Therefore, there is an urgent need for developing alternate therapeutic strategies for treating NTHi infections. Previous studies have shown that NTHi upregulates MUC5AC transcription via Toll-like receptor- (TLR-) dependent activation of p38 MAPK and transcription factor AP-1 [10]. Due to the involvement of p38 MAPK in multiple cellular processes, therapies inhibiting it can have detrimental effects in the long-term. Thus identification of novel therapeutic strategies with minimal side effects is strongly desired.

Curcumin, a yellow pigment derived from the rhizome* Curcuma longa*, is reported to possess a broad range of pharmacological effects, including antioxidant, antitumor, anti-inflammatory, antimicrobial, and antidiabetic properties [11]. Curcumin does not present a dose-limiting toxicity, thereby potentiating long-term usage with minimal side effects [12]. Curcumin is classified as “generally recognized as safe (GRAS)” by the United States Food and Drug Administration. Despite its pleiotropic effects on a multitude of diseases, poor bioavailability presents a major limitation for curcumin usage [12]. We recently reported the inhibitory effect of curcumin on NTHi-induced neutrophil recruitment in a mouse model of OM [13]. However, the effect of curcumin on regulating MUC5AC mucin, a major contributor of OM pathology, remains to be evaluated.

In this study, we demonstrate that curcumin inhibits NTHi-induced MUC5AC expression in vitro and in vivo in middle ear epithelial cells. Curcumin inhibited MUC5AC expression via inhibition of p38 MAPK via induction of negative regulator, MKP-1. Thus, our study provides evidence for the anti-inflammatory potential of curcumin in treating NTHi-induced OM by suppressing MUC5AC mucin overproduction.

## 2. Materials and Methods

### 2.1. Reagents and Antibodies

Curcumin and Actinomycin D were purchased from Sigma. SB203580 was purchased from Enzo Life Sciences. Antibodies for MUC5AC (sc-21701) and MKP-1 (sc-370) were purchased from Santa Cruz Biotechnology. Anti-mouse HRP-linked antibody (#7076) was purchased from Cell Signaling Technology. Anti-mouse Alexa 488-conjugated antibody (A11029) and anti-rabbit Alexa 488-conjugated antibody (A21206) were purchased from Life Technologies.

### 2.2. Cell Culture

Human middle ear epithelial cells (HMEECs) were grown and maintained in DMEM (Cellgro) supplemented with BEGM SingleQuots (Lonza), 10% fetal bovine serum, and 100 U/ml penicillin and 100 *μ*g/ml streptomycin (Gibco) in a humidified 5% CO2 atmosphere at 37°C.

### 2.3. Bacterial Strains and Culture Conditions

Clinical isolates of NTHi strains 12, 2627, and 9274 were used for this study [14, 15]. NTHi was prepared as described previously [13]. For in vitro experiments NTHi was resuspended in DMEM and used at a multiplicity of infection (MOI) of 50. For in vivo experiments, NTHi was resuspended in isotonic saline and used at a concentration of 5 × 10^7^ CFU per mouse. For inhibition studies, cells were pretreated with curcumin for 1 h prior to NTHi stimulation. For posttreatment studies, cells were treated with curcumin 1 h after NTHi stimulation.

### 2.4. Plasmids, Transfection, and Luciferase Assay

The expression plasmids, for constitutively active (CA) forms of MKK3 (MKK3b (E)) and MKK6 (MKK6b (E)) and dominant-negative mutant forms of p38*α* (fp38*α* (AF)) and p38*β* (fp38*β*2 (AF)), have been described previously [16]. MUC5AC-Luc luciferase reporter vector and AP-1 mutants of MUC5AC-Luc luciferase reporter vectors have been described previously [17]. Myc-MKP-1 overexpression plasmid has been described previously [13]. All transient transfections were performed using TransIT-LT-2020 transfection reagent (Mirus) according to the manufacturer's protocol. Cells were assayed 48 h after transfection. Empty vector was transfected as a control. pRL-*Renilla* luciferase vector was from Promega. Luciferase activity was measured using Dual-Luciferase Reporter Assay System (Promega). MUC5AC luciferase activity was normalized to* Renilla* activity.

### 2.5. RNA-Mediated Interference

The human pSUPER-shMKP-1 knockdown construct as described previously [18] was transfected using TransIT-LT-2020 transfection reagent (Mirus) according to manufacturer's protocol. Cells were assayed 48 h after transfection.

### 2.6. Real-Time Quantitative PCR (Q-PCR) Analysis

Total RNA was extracted with TRIzol reagent, according to manufacturer's protocol (Life Technologies). TaqMan reverse transcription reagents were used to perform reverse transcription reaction (Applied Biosystems). Real-time quantitative PCR reactions were performed using Fast SYBR Green Master Mix (Applied Biosystems) and amplified and quantified with StepOnePlus Real-Time PCR system (Applied Biosystems). Relative quantities of mRNAs were calculated using the comparative Ct method and were normalized to control, human Cyclophilin or mouse glyceraldehyde-3-phosphate (GAPDH). Human (h) and mouse (m) primer sequences for hMUC5AC, hMKP-1, hCyclophilin, mMUC5AC, and mGAPDH primer sequences were previously described [18].

### 2.7. Enzyme-Linked Immunosorbent Assay (ELISA)

HMEECs were stimulated with NTHi for 12 h. Cell culture media were harvested and centrifuged at 12,000 ×g for 10 min to precipitate cell debris. Supernatants were assayed by direct ELISA method as described previously [19]. OD was measured using Benchmark Plus microplate spectrophotometer. MUC5AC protein concentration in the supernatant was determined by normalizing to the control group.

### 2.8. Immunofluorescence Staining

Cells were grown on 18 mm round glass coverslips (VWR). Following NTHi stimulation, cells were fixed with 4% paraformaldehyde, followed by incubation with primary antibodies at 1 : 100–1 : 400 dilutions. Alexa-conjugated secondary antibody was used to detect the primary antibody. The coverslips were mounted onto glass slides using VECTASHIELD HardSet Antifade Mounting Medium with DAPI (Vector). Images were recorded with fluorescence microscopy system (BZ-X710 Microscope and BZ-X Viewer, BZ-X Analyzer imaging system, Keyence).

### 2.9. Mice and Animal Experiments

C57BL/6 mice (Jackson Laboratories) were employed. Anesthetized mice were inoculated with NTHi via the transtympanic route. For inhibition studies, mice were injected intraperitoneally (i.p) with curcumin (50 mg/kg) 1 h prior to or 1 h after NTHi inoculation. Total RNA was extracted from the dissected mice middle ear. All animal experiments were carried out following the guidelines approved by The Institutional Animal Care and Use Committee at Georgia State University.

### 2.10. In Vivo Immunofluorescence Assay

For immunofluorescence assay, formalin-fixed paraffin-embedded mouse middle ear tissues were sectioned at 4 *μ*m thickness and mounted onto glass slides. MUC5AC protein was detected using mouse anti-MUC5AC primary and Alexa 488-conjugated goat anti-mouse secondary antibodies as described previously [18, 19]. The coverslips were mounted onto glass slides using VECTASHIELD HardSet Antifade Mounting Medium with DAPI (Vector). Images were recorded with fluorescence microscopy system (BZ-X710 Microscope and BZ-X Viewer, BZ-X Analyzer imaging system, Keyence).

### 2.11. Statistical Analysis

All experiments were repeated at least three independent times. Data are represented as mean standard deviation (s.d.). Statistical significance was assessed with unpaired student's *t*-test for data with two conditions (*k* = 2) and ANOVA (followed by Tukey's post hoc) for data with more than two conditions (*k* > 2), using SPSS 22 statistics software (IBM). ^*∗*^*p* < 0.05 was considered statistically significant.

## 3. Results and Discussion

### 3.1. Curcumin Suppresses NTHi-Induced MUC5AC Expression in Middle Ear Epithelial Cells In Vitro and In Vivo

**Figure 1:**
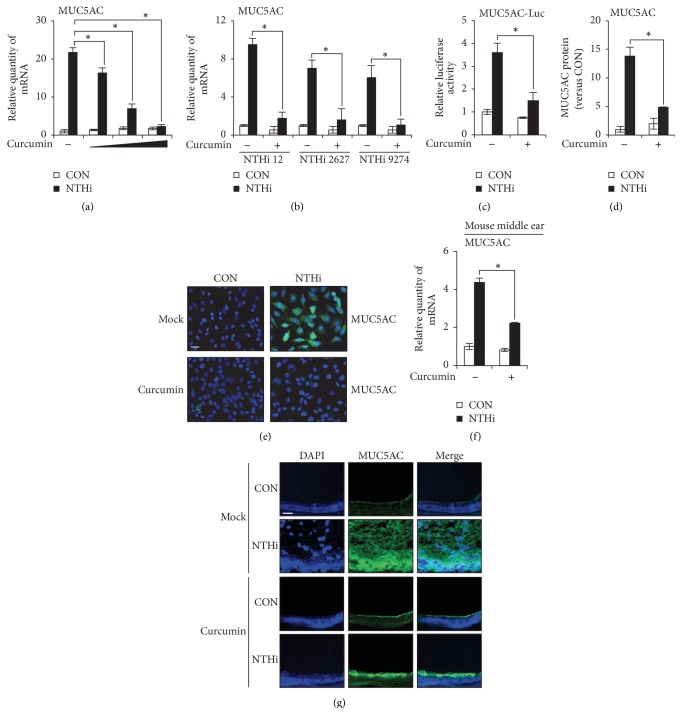
Curcumin suppresses NTHi-induced MUC5AC expression in middle ear epithelial cells in vitro and in vivo. (a) HMEECs were pretreated with 10, 20, or 50 *μ*M curcumin for 1 h, followed by NTHi for 5 h, and MUC5AC mRNA expression was measured. (b) HMEECs were pretreated with 20 *μ*M curcumin for 1 h, followed by NTHi strain 12, 2627, or 9274 for 5 h, and MUC5AC mRNA expression was measured. (c) HMEECs were transfected with MUC5AC promoter-driven luciferase vector. Cells were pretreated with 20 *μ*M curcumin for 1 h, followed by stimulation with NTHi for 5 h. MUC5AC promoter-driven luciferase activity was measured. (d) HMEECs were pretreated with 20 *μ*M curcumin for 1 h, followed by stimulation with NTHi for 12 h, and MUC5AC protein level in cell culture supernatants was measured by ELISA. (e) HMEECs were pretreated with 20 *μ*M curcumin for 1 h, followed by NTHi stimulation for 12 h. MUC5AC protein (Alexa 488) was visualized by immunofluorescence staining. DAPI, nuclear stain. Original magnification: 400x. Scale bar, 20 *μ*m. (f, g) Mice were given 50 mg/kg (i.p) curcumin 1 h prior to transtympanic inoculation of NTHi. (f) MUC5AC mRNA expression in dissected middle ear was measured after 6 h NTHi inoculation. (g) MUC5AC protein (Alexa 488) expression in dissected middle ear was visualized by immunofluorescence assay after 9 h NTHi inoculation. DAPI, nuclear stain. Original magnification: 400x. Scale bar, 20 *μ*m. Data are mean ± s.d. (*n* = 3). (a) ^*∗*^*p* < 0.05, ANOVA (Tukey's post hoc). (b)–(d), (f) ^*∗*^*p* < 0.05, *t*-test. Data are representative of three or more independent experiments.

Curcumin has been reported to suppress neutrophil migration, an early inflammatory response to NTHi infection in OM model [13]. To further evaluate the anti-inflammatory potential of curcumin in treating OM, we determined the effect of curcumin on MUC5AC mucin expression, a major contributor of OM pathogenesis. Curcumin pretreatment inhibited NTHi-induced MUC5AC mRNA expression in a dose-dependent manner in HMEECs (Figure 1(a)). Curcumin also inhibited MUC5AC mRNA expression in HMEECs stimulated with other common OM-causing NTHi strains 2627 and 9274 (Figure 1(b)), suggesting the generalizability of the inhibitory effect of curcumin on MUC5AC mucin. We next determined if curcumin inhibits MUC5AC transcription by determining the activity of MUC5AC promoter-driven luciferase vector. Curcumin suppressed NTHi-induced MUC5AC transcription (Figure 1(c)). The inhibitory effect of curcumin on MUC5AC protein levels was also confirmed by ELISA (Figure 1(d)) and by immunofluorescence staining (Figure 1(e)). Consistent with in vitro findings, curcumin pretreatment inhibited MUC5AC mRNA expression in the middle ear of mice inoculated with NTHi (Figure 1(f)). Curcumin also suppressed MUC5AC protein expression in mouse middle ear tissues (Figure 1(g)). Therefore, these data suggest that curcumin inhibits NTHi-induced MUC5AC mucin expression in middle ear epithelial cells in vitro and in vivo.

### 3.2. Curcumin Suppresses NTHi-Induced MUC5AC Expression via Inhibition of p38 MAPK

**Figure 2:**
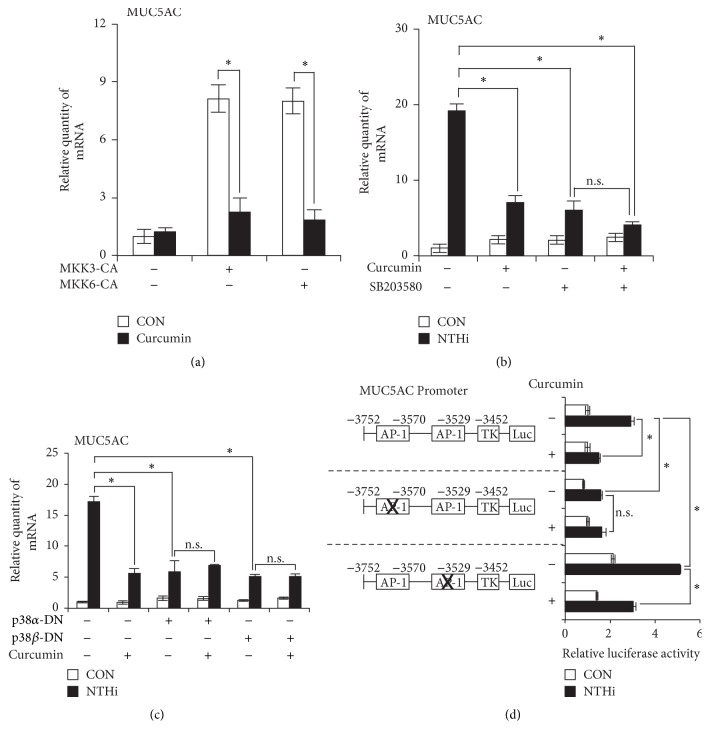
Curcumin suppresses NTHi-induced MUC5AC expression via inhibition of p38 MAPK. (a) HMEECs were transfected with Mock, MKK3-CA, or MKK6-CA plasmids. Cells were treated with 20 *μ*M curcumin for 1 h, and MUC5AC mRNA expression was measured. (b) HMEECs were pretreated with 20 *μ*M curcumin and 10 *μ*M SB203580 for 1 h, followed by stimulation with NTHi for 5 h, and MUC5AC mRNA expression was measured. (c) HMEECs were transfected with Mock, p38*α*-DN, or p38*β*-DN plasmids. Cells were pretreated with curcumin (20 *μ*M) for 1 h, followed by NTHi stimulation for 5 h and MUC5AC mRNA expression was measured. (d) HMEECs were transfected with wild-type MUC5AC promoter, distal AP-1 mutant MUC5AC promoter, or proximal AP-1 mutant MUC5AC promoter containing luciferase vectors. Cells were pretreated with 20 *μ*M curcumin for 1 h, followed by NTHi stimulation for 5 h. MUC5AC promoter-driven luciferase activity was measured. Data are mean s.d. (*n* = 3). (a) ^*∗*^*p* < 0.05, *t*-test. (b)–(d) ^*∗*^*p* < 0.05, ANOVA (Tukey's post hoc). n.s., not significant. Data are representative of three or more independent experiments.

Next, we sought to determine the mechanism by which curcumin inhibits MUC5AC expression. p38 MAPK signaling pathway has been shown to be critical in mediating NTHi-induced inflammatory responses. We previously reported that NTHi upregulates MUC5AC expression via activation of p38 MAPK [10]. In a recent study, we showed that curcumin inhibits NTHi-induced p38 phosphorylation [13]. Thus, we determined if curcumin also inhibits MUC5AC expression via suppression of p38 activation. MAPK kinases MKK3 and MKK6 are upstream activators of p38 MAPK. MKK3 activates p38*α* only, whereas MKK6 activates both p38*α* and p38*β* isoforms. Overexpression of constitutively active (CA) forms of MKK3 (MKK3-CA) or MKK6 (MKK6-CA) markedly induced MUC5AC expression, while curcumin suppressed MKK3-CA or MKK6-CA-induced MUC5AC expression (Figure 2(a)). To determine if curcumin inhibits MUC5AC expression via suppression of p38, we used multiple approaches. Pretreatment with curcumin or SB203580 (a specific inhibitor of p38 activation) alone markedly suppressed NTHi-induced MUC5AC expression in HMEECs. However, cotreatment of curcumin along with SB203580 did not further suppress MUC5AC expression (Figure 2(b)). Consistent with the p38 inhibitor data, curcumin failed to further suppress NTHi-induced MUC5AC expression in HMEECs transfected with dominant-negative (DN) mutant forms of p38 : p38*α*-DN and p38*β*-DN (Figure 2(c)).

AP-1 is one of the major transcription factors downstream of p38 MAPK. Sequence analysis of MUC5AC promoter region revealed the presence of two AP-1 transcription factor binding sites located between base pairs −3576/−3570 and −3535/−3529. Selective mutagenesis of these sites showed that the distal AP-1 site (−3576/−3570) is necessary for upregulation of MUC5AC transcription, while the proximal AP-1 site (3535/−3529) is involved in the negative regulation of MUC5AC transcription [17]. Therefore, to further determine which of these AP-1 sites is involved in curcumin-mediated inhibition of MUC5AC transcription, we employed wild-type MUC5AC promoter, distal AP-1 mutant MUC5AC promoter, and proximal AP-1 mutant MUC5AC promoter containing luciferase vectors. Curcumin pretreatment suppressed NTHi-induced transcription of both wild-type and proximal AP-1 mutant MUC5AC promoter constructs, while curcumin did not further inhibit transcription of distal AP-1 mutant MUC5AC promoter (Figure 2(d)). These findings suggest that curcumin inhibits MUC5AC transcription via inhibiting the positive AP-1 (distal site) pathway. Together these data suggest that curcumin suppresses NTHi-induced MUC5AC expression via inhibition of MKK3/6-dependent activation of p38 MAPK.

### 3.3. Curcumin Inhibits NTHi-Induced MUC5AC Expression via Upregulation of MKP-1 Phosphatase

**Figure 3:**
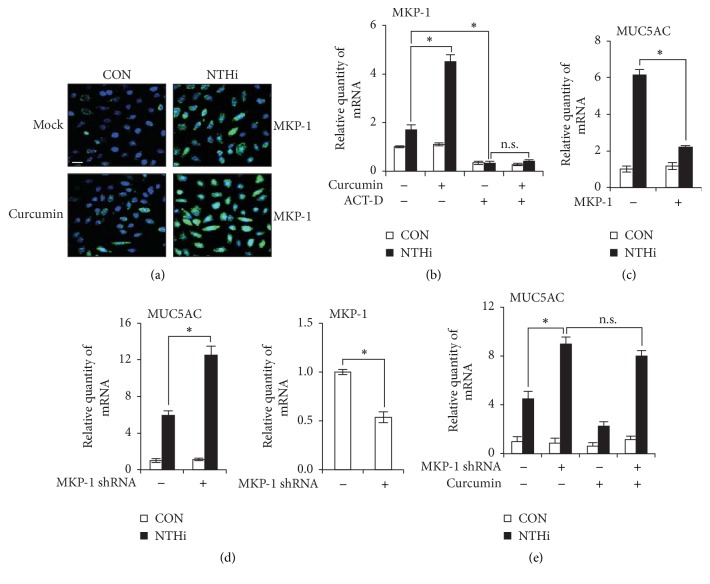
Curcumin inhibits NTHi-induced MUC5AC expression via upregulation of MKP-1 phosphatase. (a) HMEECs were pretreated with curcumin (20 *μ*M) for 1 h; followed by NTHi stimulation for 1 h. MKP-1 protein (Alexa 488) was visualized by immunofluorescence staining. DAPI, nuclear stain. Original magnification: 400x. Scale bar, 20 *μ*m. (b) HMEECs were treated with 20 *μ*M curcumin, 5 *μ*g/ml Actinomycin D (ACT-D), or both for 1 h, followed by stimulation with NTHi for 1 h, and MKP-1 mRNA expression was measured. (c, d) HMEECs were transfected with (c) Mock or myc-MKP-1, (d) Mock or MKP-1 shRNA. Cells were stimulated with NTHi for 5 h, and MUC5AC mRNA expression was measured. MKP-1 knockdown was confirmed by Q-PCR. (e) HMEECs were transfected with Mock or MKP-1 shRNA plasmid. Cells were pretreated with 20 *μ*M curcumin for 1 h, followed by NTHi stimulation for 5 h, and MUC5AC mRNA expression was measured. (b, e) ^*∗*^*p* < 0.05, ANOVA (Tukey's post hoc). (c, d) ^*∗*^*p* < 0.05, *t*-test. n.s., not significant. Data are representative of three or more independent experiments.

MAP Kinase Phosphatase-1 (MKP-1) has been shown to be a key negative regulator of inflammation via dephosphorylation of MAPKs. We previously reported that MKP-1 is a negative regulator of NTHi-induced MUC5AC expression via inhibition of p38 MAPK [20]. We previously demonstrated that curcumin markedly enhanced NTHi-induced MKP-1 expression at mRNA and protein levels. The same study showed that curcumin suppressed p38 MAPK phosphorylation via upregulation of MKP-1 expression [13]. Therefore, it is likely that curcumin suppresses NTHi-induced MUC5AC expression via a similar mechanism. We first confirmed that curcumin increases NTHi-induced MKP-1 expression by immunofluorescence staining (Figure 3(a)). To evaluate if the enhanced level of MKP-1 protein in the presence of curcumin is a consequence of increased MKP-1 transcription, we employed Actinomycin D (ACT-D), a transcription inhibitor. Pretreatment with ACT-D drastically decreased NTHi-induced MKP-1 mRNA expression. Also, in the presence of ACT-D, curcumin could not enhance MKP-1 mRNA expression upon NTHi stimulation (Figure 3(b)). These results suggest that curcumin increases MKP-1 expression via transcriptional activation. Next, we confirmed the role of MKP-1 in MUC5AC regulation. MKP-1 overexpression suppressed NTHi-induced MUC5AC expression (Figure 3(c)). Knockdown of endogenous MKP-1 with MKP-1 shRNA enhanced MUC5AC expression (Figure 3(d)). Since curcumin upregulates MKP-1 expression and MKP-1 is a negative regulator of MUC5AC expression, we sought to determine the role of MKP-1 in curcumin-mediated MUC5AC suppression. Depletion of MKP-1 with shMKP-1 rendered curcumin treatment ineffective in inhibiting NTHi-induced MUC5AC mRNA expression (Figure 3(e)). Together, these data suggest that curcumin inhibits NTHi-induced MUC5AC expression via an MKP-1 dependent mechanism.

### 3.4. Postinfection Administration of Curcumin Inhibits MUC5AC Expression in Middle Ear Epithelial Cells In Vitro and In Vivo

**Figure 4:**
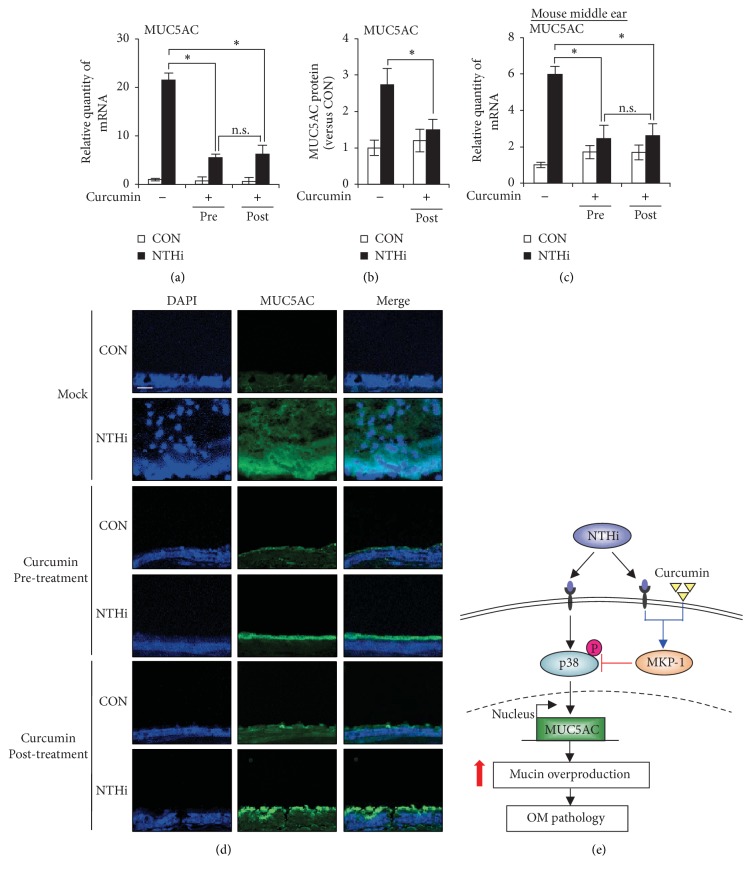
Postinfection administration of curcumin inhibits MUC5AC expression in middle ear epithelial cells in vitro and in vivo. (a) HMEECs were pretreated with curcumin (20 *μ*M) 1 h prior to NTHi stimulation or posttreated with curcumin (20 *μ*M) 1 h after NTHi stimulation. MUC5AC mRNA expression was measured after 5 h NTHi stimulation. (b) HMEECs were posttreated with 20 *μ*M curcumin 1 h after NTHi stimulation. 12 h after NTHi stimulation, MUC5AC protein levels in cell culture supernatants were measured by ELISA. (c, d) Mice were given 50 mg/kg (i.p) curcumin 1 h prior to transtympanic inoculation of NTHi or were given 50 mg/kg (i.p) curcumin 1 h after NTHi inoculation. (c) MUC5AC mRNA expression in dissected middle ear was measured after 6 h NTHi inoculation. (d) MUC5AC protein (Alexa 488) expression in dissected middle ear was visualized by immunofluorescence assay after 9 h NTHi inoculation. DAPI, nuclear stain. Original magnification: 400x. Scale bar, 20 *μ*m. (e) Schematic representation illustrating that curcumin suppresses NTHi-induced MUC5AC expression via MKP-1-dependent inhibition of p38 MAPK. (a, c) ^*∗*^*p* < 0.05, ANOVA (Tukey's post hoc). (b) ^*∗*^*p* < 0.05, *t*-test. n.s., not significant. Data are representative of three or more independent experiments.

Next, we sought to evaluate the therapeutic relevance of the inhibitory effect of curcumin on MUC5AC mucin in an NTHi-induced OM setting. Thus, we evaluated the effect of administering curcumin after NTHi infection, which resembles a clinically relevant setting. Curcumin administration after NTHi infection significantly suppressed NTHi-induced MUC5AC expression at mRNA (Figure 4(a)) and protein levels (Figure 4(b)) in vitro. Consistent with in vitro findings, curcumin treatment after NTHi infection inhibited MUC5AC mRNA (Figure 4(c)) and protein expression (Figure 4(d)) in mouse model for OM. Curcumin suppressed MUC5AC expression to the same extent under both pre-NTHi and post-NTHi infection conditions. Thus, these data suggest that curcumin is a potential therapeutic for treating NTHi-induced MUC5AC mucin overproduction as seen in OM.

Here we show that curcumin exerts its anti-inflammatory effects by increasing MKP-1 transcription. Increased MKP-1 transcription thereby enhances MKP-1 protein levels, which leads to p38 MAPK inhibition. Further studies are needed to identify the transcriptional factors, transcriptional machinery involved in curcumin-mediated MKP-1 induction. Previously it has been demonstrated that curcumin enhanced the phosphorylation and activation of MKP-1, leading to p38 MAPK inhibition [21]. Therefore, it is likely that curcumin modulates the activity of negative regulator MKP-1 via transcriptional, translational, and posttranslational mechanisms. Understanding the specific roles of these modulations and the specific signaling molecules involved could help identify novel drug targets for curcumin to counteract overactive immune responses.

In the current study curcumin was administered via intraperitoneal (i.p) injection. Systemic oral administration of antibiotics is the most common clinical strategy for treating OM. It is likely that systemic i.p administration of curcumin could have exerted global effects on the body including the middle ear. Our previous studies evaluating the effect of inhibitors such as rolipram, roflumilast, and vinpocetine on mucin expression in bacteria-induced OM model have also used i.p injection as the route of administration [18, 19]. Since curcumin is known for its safety and tolerability in patients, even with prolonged usage, it is likely that the systemic effects of curcumin would not be detrimental. However, to design therapies with increased specificity further studies focusing on evaluating the therapeutic efficiency of ototopical application of curcumin in resolving OM are needed. Further studies targeted towards increasing the bioavailability and development of ototopical drug delivery systems will be of clinical significance in treating OM. The insights of this study may have broader applications in the context of other chronic inflammatory conditions.

## 4. Conclusions

In summary, our study demonstrates that curcumin inhibits NTHi-induced MUC5AC expression in vitro and in vivo. Curcumin suppressed NTHi-induced MUC5AC expression by inhibition of p38 MAPK via upregulation of MKP-1 (Figure 4(e)). Curcumin treatment after NTHi infection also inhibited MUC5AC expression in a mouse model of OM, suggesting the clinical relevance of our findings. Thus, our study reports for the first time the efficacy of curcumin in treating NTHi-induced MUC5AC mucin overproduction.
